# ANXA6: a key molecular player in cancer progression and drug resistance

**DOI:** 10.1007/s12672-023-00662-x

**Published:** 2023-05-02

**Authors:** Jinlong Cao, Shun Wan, Siyu Chen, Li Yang

**Affiliations:** 1 Department of Urology, Lanzhou University Second Hospital, Lanzhou, 730000 China; 2 Gansu Province Clinical Research Center for Urology, Lanzhou, 730000 China

**Keywords:** ANXA6, Migration, Drug resistance, Metabolic reprogramming, Membrane repair

## Abstract

Annexin-A6 (ANXA6), a Ca^2+^-dependent membrane binding protein, is the largest of all conserved annexin families and highly expressed in the plasma membrane and endosomal compartments. As a multifunctional scaffold protein, ANXA6 can interact with phospholipid membranes and various signaling proteins. These properties enable ANXA6 to participate in signal transduction, cholesterol homeostasis, intracellular/extracellular membrane transport, and repair of membrane domains, etc. Many studies have demonstrated that the expression of ANXA6 is consistently altered during tumor formation and progression. ANXA6 is currently known to mediate different patterns of tumor progression in different cancer types through multiple cancer-type specific mechanisms. ANXA6 is a potentially valuable marker in the diagnosis, progression, and treatment strategy of various cancers. This review mainly summarizes recent findings on the mechanism of tumor formation, development, and drug resistance of ANXA6. The contents reviewed herein may expand researchers’ understanding of ANXA6 and contribute to developing ANXA6-based diagnostic and therapeutic strategies.

## Introduction

Annexins, a superfamily of secreted proteins in the cytoplasm and attached to the phospholipid membrane of cells, are highly conserved Ca^2+^-dependent membrane-binding proteins [1]. Annexin was first discovered and purified by Creutz et al. [2]. Since the discovery of the first human annexin A1 and A2 [3], more than 1000 members of the annexin subfamily have been identified. These annexins are classified into five groups (Groups A–E) and widely distributed in various tissues and cells of animals and plants. Only 13 members of group A (annexin A1–A13) were found in human organs [4]. Annexin involves various cellular functions, mainly vesicle trafficking and membrane repair [1, 5]. Many studies have shown that annexins, especially ANXA6, play an important role in tumor formation, development, and drug resistance [6, 7].

ANXA6 is located on chromosome 5q32-q34, and the coding gene is approximately 60 kbp in base length, containing 26 exons and encoding a protein of about 68 kDa [6, 8]. The C-terminus of ANXA6 contains two annexin domains (probably evolved from the fusion copy of the ANXA5 and ANXA10 genes), composed of eight repeats of 70 amino acid sequences [1, 8]. These repeat sequences are packed into a disk, which is mostly α-helical with a slight curvature [8]. When the ANXA6 C-terminal binds to the phospholipid membrane, the flexible N-terminal domain is located in the concave face of annexin to mediate regulatory interactions with protein ligands and regulate annexin-membrane association [9, 10]. The diversity of the N-terminal domain is also the classification criterion for distinguishing different subfamilies of annexins.

ANXA6 is the largest protein of all conserved annexins and is found primarily in the plasma membrane and endosomal compartments. ANXA6 acts as a multifunctional scaffold protein, recruiting signaling proteins, regulating cholesterol and membrane transport and influencing actin dynamics [11–15]. ANXA6 binds to specific membrane phospholipids in a Ca^2+^-dependent manner, providing a link between Ca^2+^ signaling and membrane function. The Ca^2+^-binding sites of ANXA6 are located in the annexin repeats 1, 2, 4, 5, 6, and 8 [16]. Ca^2+^-activated ANXA6 binds to negatively charged phospholipids [8, 17], which can allow it to participate in the composition and organization of cytoplasmic, cytoskeletal and cellular membrane [10]. Although ANXA6 lacks enzymatic activity, it can mediate a number of functions involving membrane, nucleotide, cholesterol binding, and scaffolding of specific proteins or multifarious protein complexes. These properties enable ANXA6 to participate in membrane dynamics-related events such as signal transduction, cholesterol homeostasis, and the formation of multifactor protein complexes and membrane domains associated with intracellular/extracellular membrane transport [8]. Thus, ANXA6 is related to many cancer-related biological processes, including vesicular transport [18, 19], cell proliferation and division, apoptosis, calcium signaling, and growth regulation, among many other cellular functions [6, 20]. Moreover, ANXA6 is differentially expressed in melanoma, cervical cancer, epithelial cancer, breast cancer, gastric cancer, and other tumors [20]. However, the specific mechanism of action of ANXA6 in different tumors depends on the corresponding tumor type and cellular characteristics.

Increasingly studies have shown that ANXA6 plays an essential role in the formation and development of various cancers and has a specific impact on various tumor-associated phenotypes, such as proliferation, migration, invasion, drug resistance, and metabolic reprogramming [6]. Investigating the precise molecular mechanism of ANXA6 and developing effective targeted drugs against ANXA6 are the most important topics in ANXA6 research. This paper presents a review of the relevant studies on the mechanism of ANXA6 in tumor development and drug resistance.

## Role in cancer: cancer promoter or cancer suppressor?

ANXA6 exhibits a dual role in different tumors, acting as a tumor suppressor or promoter depending on the cancer type and malignancy. ANXA6 is down-regulated in many cancers, and acts as cancer suppressor, which include highly aggressive triple-negative breast cancer (TNBC) subtypes [21, 22], gastric cancer [23], cervical cancer [24], and hepatocellular carcinoma [25], bladder cancer [26]. However, elevated ANXA6 levels have been found in the progression of pancreatic cancer [27–29], female thyroid cancer [30], squamous cervical cancer [31], ovarian cancer [32], esophageal adenocarcinoma [33], and melanoma [34]. The expression of ANXA6 in various tumors is presented in Table 1. These evidences suggest that ANXA6 is differently expressed in many cancers and shows a high potential value in the diagnosis, prognosis, and treatment strategies of various cancers.

**Table 1:** the expression of ANXA6 in multiple cancers

Cancer type	ANXA6 expression description	Detection	Marker	References
Breast cancer	Significantly associated with the survival of patients with basal-like breast cancer	WB	Predictive biomarker for basal-like breast cancer patient survival	[22]
Breast cancer	Reduced expression in breast cancer tissues, but elevated in invasive breast cancer phenotypes	WB	Biomarker for invasive breast cancer phenotypes	[44]
Gastric cancer	ANXA6 was down-regulated in gastric cancer cells and primary gastric carcinomas	PCR	ANXA6 methylation is not a prognostic factor	[23]
Hepatocellular carcinoma	Downregulated in human hepatocellular carcinoma	WB	Diagnosis marker	[25]
Bladder cancer	Significantly decreased in bladder tumor vs. normal tissues	Bioinformatic transcriptome	Marker for subtype classification	[26]
Pancreatic cancer	High expression in pancreatic cancer and lung squamous cancer vs. normal tissues	IHC	MAb9E1 can as a therapeutic option	[29]
Female thyroid cancer	Positive association between blood BPA levels and mRNA expression of the ANXA6 and VCP	2-DE, PCR	ANXA6 and VCP are proteomic biomarkers for BPA-early life exposure	[30]
Cervical squamous cancer	Increased in cervical intraepithelial neoplasia and microinvasive cervical cancer vs. squamous cervical cancer precursor lesions	2-DE, IHC	Diagnosis of cervical cancer progression	[24, 31]
Ovarian cancer	Markedly increased in advanced-stage tumors vs. benign	2-DE, WB, ELISA	Diagnosis marker of advanced ovarian cancer stages	[32]
Esophageal adenocarcinoma	AnxA6 is a component of a 4-protein serum biomarker panel	Proteomics, ELISA	Noninvasive detection of early tumor stages in patient serum	[33]
Melanoma	Decrease or loss of Annexin VI expression as melanomas progressed from a benign to a more malignant phenotype	Northern blotting	A potential role of Annexin VI in tumor suppression	[34]
Acute myeloid leukemia	ANXA6 was significantly associated with worse prognosis	DNA Microarray	A biological marker for prognosis of pediatric AML	[51]
Acute lymphoblastic leukemia	Highly expressed in B-lineage acute lymphoblastic leukemia vs. normal B-cell progenitors	cDNA array analysis	Diagnosis of B-lineage ALL	[52]

**Fig. 1:**
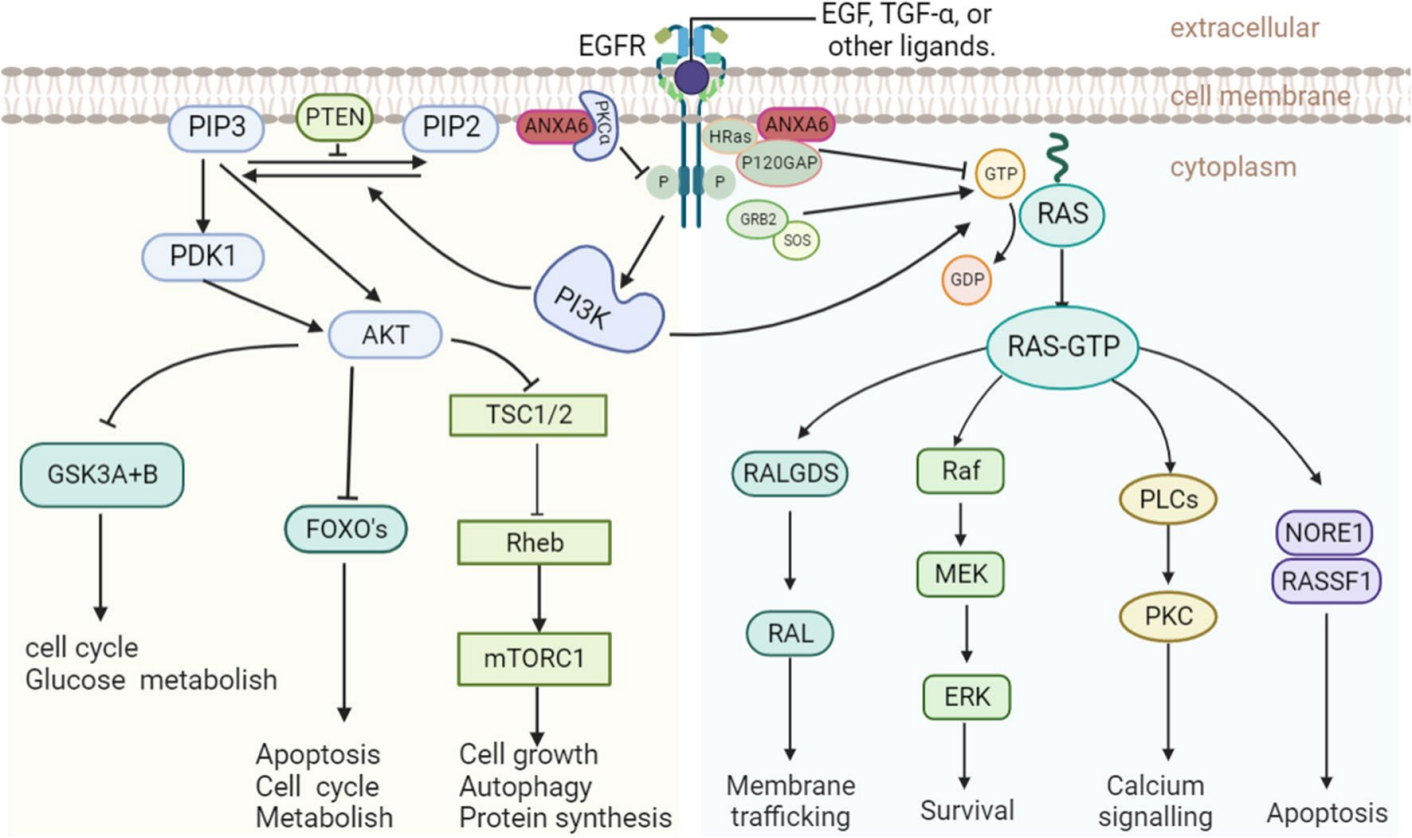
ANXA6 may affect the downstream pathway by inhibiting EGFR phosphorylation. ANXA6, as a scaffold protein of Protein kinase Cα (PKCα), PKCα phosphorylates EGFR at threonine 654 to inhibit EGFR tyrosine phosphorylation and related activation of downstream effectors. Moreover, ANXA6 forms protein complexes with H-Ras, p120GAP, and EGFR that inhibit p120GAP/Ras assembly and inhibit Ras signaling

ANXA6 is generally considered a tumor suppressor gene, mainly because ANXA6 negatively regulates epidermal growth factor receptor (EGFR) phosphorylation and its downstream the Ras-Raf-mitogen activated protein kinases (MAPK) and phosphatidylinositol-3-kinase/Akt (PI3K-AKT) pathway, which are two important signals for tumorigenesis, thereby affecting a variety of tumor-associated phenotypes, as is shown in the Fig. 1 [35–38]. In both vitro and vivo models, there is direct connection between ANXA6 and p120 GTPase activating protein (p120GAP) [37, 39]. Moreover, cell-experiments demonstrate that ANXA6 promotes membrane binding of p120GAP in a Ca^2+^-dependent manner [37, 38]. Targeting p120GAP to the plasma membrane can promote the formation of the Ras-p120GAP complex and further reduce the level of active Ras and the activity of the pathway [39–43], and further inhibits the oncogenic potential of tumor growth and movement [11, 36]. The scaffolding and auxiliary functions of ANXA6 inhibition of oncogenic signaling cascades are potentially valuable for malignant tumor development [20, 44].

ANXA6 expression of breast cancer appears to be complex and multifunctional. Reduced ANXA6 expression was found in breast cancer tissues, but elevated in invasive breast cancer phenotypes [44]. Low ANXA6 expression was also significantly associated with suppressed tumor activity and the survival of patients with basal-like breast cancer [21, 22]. ANXA6 is downregulated in many EGFR (+) and estrogen receptor (ER) (–) breast cancer cells [37]. ANXA6 knockdown in breast cancer cells (MDA-MB-436) increased Ras activity and cell proliferation in anchored growth assay [37]. Francia et al. [34] found that ANXA6 mRNA and protein levels were lower in mouse metastatic melanoma B16F1O cells than in syngeneic immortalized melanoma cells and that expression levels were negatively correlated with melanoma progression. Upregulation of ANXA6 promotes protein kinase Cα (PKCα)-mediated EGFR phosphorylation, thereby inhibiting EGFR tyrosine kinase activity. This is associated with reduced growth in squamous cell carcinomas with elevated ANXA6 as well as decreased wound healing, stroma and organ-type stroma invasiveness [45]. In addition, increased ANXA6 protein levels contribute to the improved efficacy of tyrosine kinase inhibitors (TKIs) targeting EGFR to reduce EGFR-induced growth, migration, and invasive properties of squamous epithelial carcinoma cells [36, 41, 45]. On the other hand, ANXA6 is downregulated in cervical cancer, and elevated ANXA6 expression may induce autophagy-related signaling to inhibit cervical cancer tumorigenesis [46]. Interestingly, the expression of ANXA6 increases from cervical intraepithelial neoplasia 2/3 (CIN2/3) to cervical micro-invasive carcinoma [24, 31]. Similarly, ANXA6 is downregulated in human hepatocellular carcinoma [25]. ANXA6 is downregulated by promoter methylation in gastric cancer [23] and overexpression of ANXA6 inhibits gastric cell proliferation [47]. Evidence from bioinformatics analysis demonstrated that ANXA6 is downregulated in bladder cancer [26].

Unlike the tumors mentioned above, ANXA6 expression is elevated or unclear in some tumors. ANXA6 is highly expressed in osteosarcoma [48], and regulates mineralization during carcinogenesis in osteosarcoma [49]. Upregulation of ANXA6 in ovarian cancer promotes cell proliferation [32]. Elevated ANXA6 was detected in the feces of colorectal cancer patients, implicating ANXA6 as a fecal biomarker for early detection of high-risk adenomas and colorectal cancer [50]. ANXA6 is also highly expressed and is beneficial for diagnosis and prognosis prediction in B-lineage acute lymphoblastic leukemia and acute myeloid leukemia [51, 52]. Although there was no difference in ANXA6 expression between cancer and para-cancer in female thyroid cancer patients, blood bisphenol A (BPA) levels were positively correlated with ANXA6 gene expression [30]. BPA is one of the risk factors that promote the development of breast cancer.

These evidences suggest that ANXA6 has different properties in different tumors. Therefore, it is necessary to understand the function and mechanism of action of ANXA6 in various tumors.

## Roles of ANXA6 in cancer migration and invasion

The ability of cancer cells to reach distant tissues through migration and invasion leads to the metastatic spread of tumors, which is the cause of death for 90% of cancer patients [53]. Interaction of the extracellular ANXA6 proteins with extracellular matrix proteins may affect adhesive and migratory cell properties [54–56]. ANXA6 provides membrane scaffold functions that lead to lipid and actin cytoskeletal rearrangement and enable recruitment of signaling proteins that facilitate adhesion assembly and signaling [15, 57–59]. Moreover, the repair ability of cell membrane damage may be the driving force of cancer cell metastasis [60–62]. Therefore, inhibition of cell membrane repair mechanisms may inhibit cancer cell metastasis [63]. ANXA6 is a multifunctional scaffold for cell movement that links to a variety of proteins in the extracellular matrix and cytoskeleton [64], and the abnormal expression may impair membrane repair processes and cancer metastasis. But in some studies, ANXA6 seems to have been the opposite effect. This may be because cell signaling crosstalk between the tumor and the tumor environment impacts its mechanism of action, such as the prominent crosstalk of de-regulated EGFR and Ras activity with integrin, focal adhesion kinase (FAK), Src, Rac/Rho GTPases signaling [43, 65–68]. ANXA6 is associated with a variety of proteins that have different effects on the migration and invasion ability of different cells. The main molecules are shown in Table 2.

**Table 2:** ANXA6 interactions relevant in cell migration and adhesion

ANXA6 interactions	Functions	Site of action	References
Glycosaminoglycans (e.g., chondroitin sulfate, heparin, heparin sulfate)	Promote cancer cell adhesion	Extracellular	[54–56]
Fetuin-A	Promote cancer cell adhesion and motility	Extracellular	[44]
S100A8, S100A9	Neutrophil chemotaxis and adhesion, inflammation-related cancers	Extracellular	[64]
LDL receptor-related protein 1 (LRP1), thrombospondin 1 (TSP1)	ANXA6/LRP1/TSP1 complexes on exosomes support pancreatic cancer aggressiveness	Extracellular	[28, 57]
Dysferlin, actin, AnxA1, A2, A5	Leakage of ANXA6 into extracellular space during membrane repair	Extracellular	[60–62]
Phospholipids and cholesterol	Sustaining a lipid raft-like membrane environment for cell adhesion and migration	Focal adhesions, lipid rafts	[57, 58]
Ca^2+^ release channel	Ca^2+^-channels to regulate Ca^2+^-influx during cell migration	Late endosomes, endoplasmic reticulum	[72, 73]
Actin	Cytoskeletal rearrangements and formation of reversible membrane-cytoskeleton complexes	Cortical cytoskeleton at cell protrusions	[15, 59]
EGFR, protein kinase Cα (PKCα)	Scaffold function: PKCα recruitment for EGFR inactivation to enable focal adhesion turnover	Plasma membrane	[36, 38]
H-Ras, p120GAP, Raf-1	Scaffold function: p120GAP recruitment for Ras inactivation	Plasma membrane	[39, 41, 42]
Pyk2, Fyn, RasGAP	A novel protein complex may well be linked with a Ca^2+^ mediated regulation of p21 Ras activity.	Unknown	[43]
LE-cholesterol transporters, other LE proteins	LE-cholesterol delivery to the Golgi, recycling endosomes and plasma membrane	LE, lysosome	[14, 38]

Breast cancer is one of the tumors with the most biological signals and target molecules in all neoplasms. The mechanism of ANXA6 has been extensively studied in breast cancer. Silencing of ANXA6 in invasive BT-549 breast cancer cells could enhance the anchor cell growth, but with strong inhibition of intercellular cohesion, cell adhesion/diffusion, cell motility, and invasiveness [44]. The specific mechanism may be the depletion of ANXA6 leads to the focal adhesion kinase and the PI3K/AKT pathway strongly inhibited while the MAPK pathway remained constitutively active [44]. In mouse TNBC xenografted models, the loss of ANXA6 is associated with tumorigenesis and development, and ANXA6 may inhibit tumor proliferation in TNBC cells [69]. That is consistent with the situation in vitro experiments. Coincidentally, the silencing of ANXA6 leads to the death of migrating breast cancer cells (MDA-MB-231) due to the double-sided nature of membrane repair mechanisms [63]. The study of O’Sullivan et al. [29] showed that MAb9E1 acts as an upstream antibody protein to ANXA6, and it can reduce the invasive ability of breast cancer by downregulating the expression of ANXA6. Indeed, the reciprocal expression of ANXA6 and general regulatory factor 2 (GRF2) can be used to delineate GRF2-low/ANXA6-high invasion from GRF2-high/ANXA6-low rapidly growing TNBC [70]. Moreover, cytotoxic drugs for breast cancer, such as taxanes and anthracyclines, could trigger the release of ANXA6-rich extracellular vesicles (ANXA6-EVs) from cancer cells, which promote tumor migration and invasion [27]. The above evidence suggests that ANXA6 may be a reliable biomarker for distant recurrence-free survival and chemoresistance in TNBC patients. For other tumor types, the silencing of ANXA6 could reduce the aggressiveness of pancreatic and lung squamous carcinoma [29]. ANXA6-EVs, derived from cancer-associated stromal fibroblasts (CAF), promotes tumor cell invasiveness in pancreatic ductal adenocarcinoma, and the blockade of CD9 impairs the uptake of ANXA6-EVs by pancreatic ductal adenocarcinoma cells [28, 71]. In human squamous epithelial carcinoma, elevated AnxA6 scaffold levels contribute to improve TKI-mediated inhibition of growth and migration, but also invasive properties in EGFR overexpressing cells [45]. In pheochromocytoma cell line P12, ANXA6 overexpression can increase intracellular Ca^2+^ levels, decrease catecholamine secretion and promote tumor migration [72]. The probable mechanism is that ANXA6 promotes Ca^2+^-release through Ca^2+^ gated channel [73]. In summary, the silencing of ANXA6 is closely associated with decreased invasiveness in a variety of tumors, which indicated that ANXA6 can be utilized to predict the invasion and migration ability of a variety of tumors.

Nevertheless, ANXA6 showed the opposite effect on several cancers. García-Melero et al. [74] found that ANXA6 overexpression in human A431 squamous cell carcinoma, hamster ovary cells, and HeLa cells could reduce migration and invasion. In terms of the mechanics, elevated ANXA6 leads to the inhibition of cholesterol export from late endosomes (LEs) and cholesterol accumulation in LEs, which inhibits Stx6-dependent integrin recycling. In addition, low-density lipoprotein (LDL), as a carrier of cholesterol, was also regulated by the cholesterol content in LEs. Overexpression of ANXA6 and accumulation of cholesterol in LEs could reduce cholesterol-sensitive cell mass formation, fibronectin (FN) secretion, and integrin recycling [18, 74, 75], and thereby reducing LDL-induced migration and invasion of hamster ovarian cells, and A431 cancer cells [45, 74, 76]. Furthermore, deficiency of ANXA6 in Niemann-Pick C1 (NPC1) mutant cells stimulates StAR-related lipid transfer domain-3 (StARD3)-dependent restoration of LE/Lys-Chol output, leading to increased amounts of cholesterol in focal adhesions and cholesteryl ester stores, which improves LDL-induced migratory activity [76]. ANXA6-regulated LDL transport routes contribute to cholesterol delivery to focal adhesion structures, thereby improving the migratory behavior of NPC1 mutant cells [76]. Therefore, ANXA6 can exert different migratory effects on the above tumors by regulating the transport of LDL and cholesterol.

## ANXA6 and cancer drug resistance

The specific intracellular and extracellular scaffolding functions of ANXA6 and its interacting proteins may contribute differently to various cancers’ progression and treatment outcomes [20, 21, 77]. Many studies have indicated that ANXA6 is associated with tumor drug resistance. The mechanisms of action involved are mainly focused on three aspects: involvement in the metabolism of cholesterol in LEs, interaction with EGFR, and participation in autophagy regulation. ANXA6 can even be used to predict cancer recurrence and chemotherapy response. Therefore, it is necessary to understand the mechanisms of ANXA6-mediated drug resistance in tumors.

### ANXA6 induces drug resistance through cholesterol metabolism

**Fig. 2:**
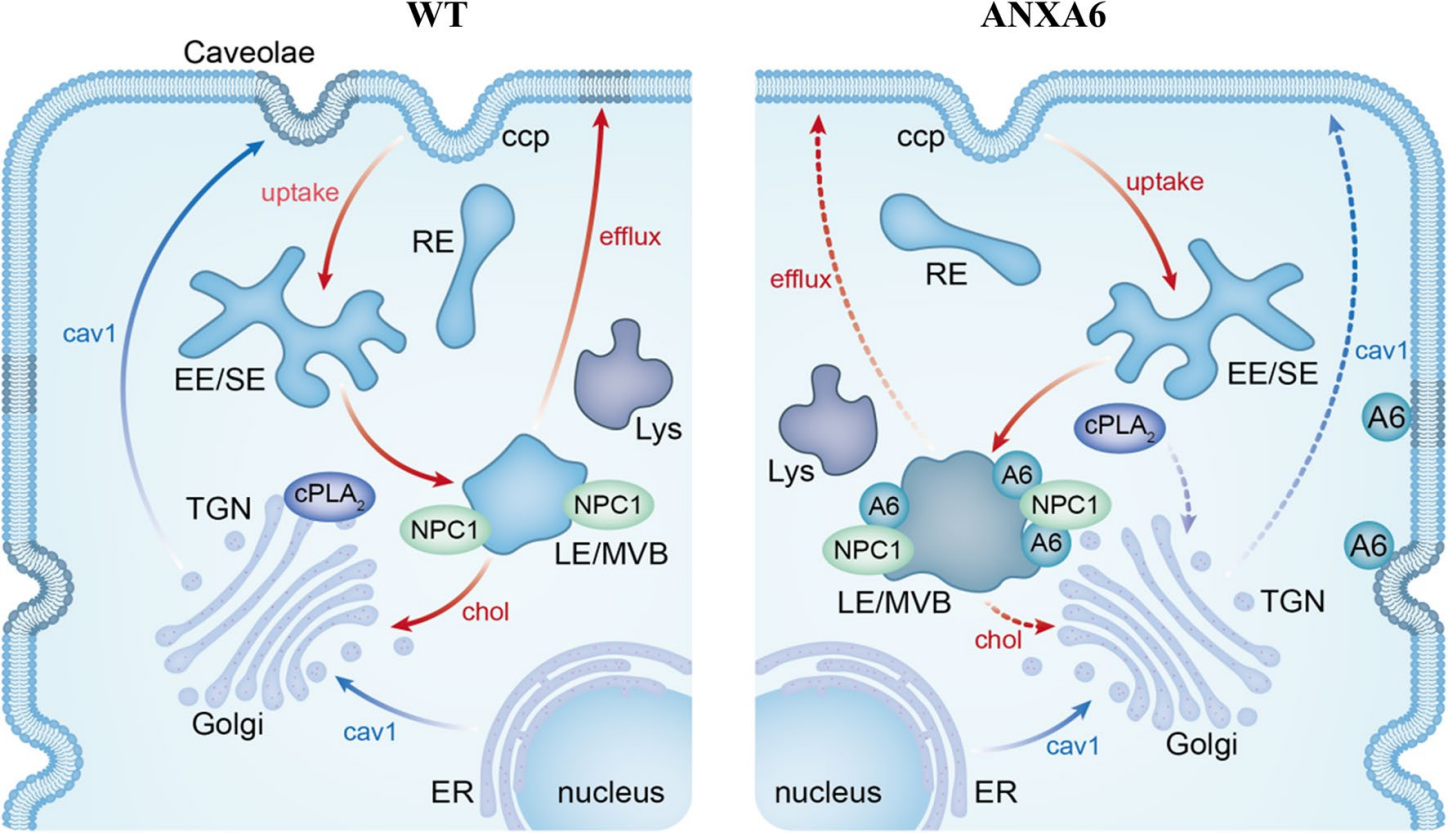
Role of ANXA6 in late endosomal (LE) and lysosome (Lys) cholesterol transport and caveolae formation. WT (low-level ANXA6) cell is shown in the left panel. The blue arrow shows the pathway of Caveolin-1 (Cav-1) from the endoplasmic reticulum (ER) to the cytomembrane. Red arrows indicate cholesterol uptake, transport from LEs to Golgi and efflux. In cells with high ANXA6 levels (right panel), cholesterol buildup in LEs is accompanied by ANXA6 translocation to LE/ multivesicular bodies (MVBs), where ANXA6 interferes with the Niemann-Pick C1 (NPC1) protein to inhibit cholesterol output to the Golgi. Excessive accumulation of LE/Lys cholesterol interferes with the transport of Cav-1 to the cell surface dependent on cytoplasmic phospholipase A2α (cPLA2α). And ultimately leads to the reduction of Cav-1 and cholesterol in the plasma membrane and subsequent caveolae formation and signaling

Reprogramming of lipid metabolism is one of the common phenomena in cancer. Metabolic adaptation due to increased cholesterol demand is a mechanism for cancer growth and development, which can eventually lead to tumor drug resistance [78, 79]. Cholesterol requirements and the expression of many LDL receptors are increased in tumor cells during drug therapy [11]. Many studies have suggested that ANXA6 expression is highly correlated with cholesterol homeostasis. As a critical factor of the LE/Lys-Chol transport channel, ANXA6 can control the adhesion of cell migration and the storage of lipid droplets [14], and the roles of ANXA6 in LE cholesterol transport are shown in Fig. 2. ANXA6 may affect the growth and invasion of a variety of tumor cells by affecting LE/Lys-Chol transport channels [18, 19, 74, 80]. ANXA6 also regulates membrane-actin interactions [81, 82] and LDL-targeting lysosomes during endocytosis transport [83, 84]. When the excessive accumulation of LE/Lys-Chol interferes with cytoplasmic phospholipase A2 (cPLA2)-dependent Caveolin-1 (Cav-1) transport to the cell surface. It ultimately leads to mislocalization and dysfunction of the SNAP receptor (SNARE) protein complexes (e.g., Stx4, SNAP23, and Stx6) [18, 74, 85]. ANXA6-regulated cholesterol transport routes from the LE/Lys channels appear to contribute to cholesterol delivery to focal adhesions, thereby improving migratory activity [76]. These molecular and metabolic changes not only lead to changes in tumor malignancy, but also change tumor drug resistance. When TNBC cells are chronically exposed to Lapatinib or other TKIs targeting EGFR, this is accompanied by ANXA6 upregulation and LE/Lys-Chol accumulation, ultimately leading to tumor resistance [21, 86]. This evidence suggests that ANXA6 levels may reflect anti-cancer drug resistance associated with cholesterol metabolic adaptation [79, 87–89]. Notably, ANXA6 depletion in NPC1 mutant cells can restore cholesterol efflux from LE/Lys in a Rab7-dependent manner [80]. ANXA6 overexpression leads to LE/Lys-Chol accumulation, similar to the loss of NPC1 function. Therefore, many molecular signals and genes are involved in ANXA6-mediated cholesterol metabolism and thus influence drug resistance, which is an urgent direction to be solved and explored.

### ANXA6 regulates drug resistance through EGFR interactions

ANXA6 is a novel PKCα and GTPase-activating protein p120 scaffold on the plasma membrane. PKCα could phosphorylate the EGFR at threonine 654 to inhibit EGFR tyrosine phosphorylation (pY-EGFR) and the associated activation of downstream effectors. As a new PKCα membrane scaffolding protein, elevated ANXA6 could promote PKCα-mediated EGFR inactivation through increased membrane targeting of PKCα and EGFR/PKCα complex formation [36, 90]. Moreover, the association of ANXA6 with H-Ras-containing protein complexes may contribute to the regulation of EGFR overexpression and p120GAP/Ras assembly in ER (−) breast cancer cells, thereby inhibiting Ras signaling in breast cancer cells [37]. Taken together, ANXA6 can down-regulates EGFR, which may inhibit its downstream PI3K/AKT and RAS signaling pathways, thereby affecting a variety of tumor-associated phenotypes, as shown in the Fig. 1. Conversely, ANXA6 could stabilize activated EGFR and activate the downstream pathway of the EGFR active signaling pathway. Co-immunoprecipitation expression (co-IP) and Glutathione-S-transferase (GST) pull-down assays indicate that ANXA6 could directly interacts with EGFR. ANXA6 can induce gemcitabine resistance by inhibiting EGFR ubiquitination and degradation in TNBC [91]. Therefore, the interaction of ANXA6 with the EGFR may be a potential mechanism for tumor drug resistance. ANXA6 modulates the cytotoxicity and efficacy of EGFR-targeted therapies in TNBC cells, ultimately leading to the development of drug resistance [22, 44, 69]. In addition, ANXA6 downregulation promotes the degradation of activated EGFR, sensitizes TNBC cells to EGFR-TKI, and is associated with poorer overall survival and distant metastasis-free survival [21, 22]. Elevated ANXA6 also improves TKI-induced anti-migration and anti-invasiveness in A431 human squamous cell cells with high EGFR expression but lacking endogenous ANXA6 [45]. The expression of ANXA6 and drug resistance can be mediated by a variety of factors. Stephen et al. [92] found that TNBC cells are exposed to hypoxia (> 24 h) can stimulate the expression of ANXA6 rather than briefly exposed (< 24 h). And hypoxia-induced ANXA6 expression could also increase the resistance of TNBC cells to EGFR and androgen receptor (AR) antagonists. Sarrah et al. [86] also found lapatinib-induced upregulation of ANXA6 and accumulation of cholesterol in LEs constitute novel resistance mechanisms to EGFR-targeted TKIs and mitigate acquired resistance to these drugs. Apart from drug and environment induced elevation of ANXA6, extracellular vesicles rich in ANXA6 can also be transferred from gemcitabine-resistant TNBC cells to sensitive cancer cells to increase ANXA6 expression. Upregulation of exosomal-ANXA6 (ANXA6-exo) enhances cell viability and colony formation and inhibits apoptosis in sensitive cancer cells [91]. Moreover, serum ANXA6-exo levels in TNBC patients may predict response to gemcitabine chemotherapy [91]. Based on the above overview, ANXA6 not only interacts directly or indirectly with EGFR, but also plays a significant role in drug resistance.

### ANXA6 induces drug resistance through the regulation of autophagy

As a membrane scaffolding protein, ANXA6 may stabilize activated cell-surface receptors to regulate cellular processes, such as regulates autophagy and endocytic transport [13]. Elevated ANXA6 has been proved to increase drug resistance by regulating autophagy in various tumors [46, 93, 94]. ANXA6-EVs induced by continuous chemotherapeutic pressure promotes drug resistance, cell migration, stemness, and autophagy in paclitaxel-sensitive breast cancer cells [95]. ANXA6-EVs up-regulate yes-associated protein (YAP) to promote dysregulation of the Hippo pathway, contributing to the development of tumor resistance to some extent. In addition, silencing YAP could counteract the effects of ANXA6-EVs on paclitaxel resistance and cancer aggressiveness in bladder cancer cells [95]. In nasopharyngeal carcinoma, ANXA6 promotes protective autophagy by inhibiting the PI3K/AKT signaling pathway, thereby promoting radioresistance [94]. For all the current evidence, elevated ANXA6 can induce protective autophagy, and enhance the resistance to chemotherapy and radiotherapy.

### Drug resistance caused by other pathways

Annexins can be involved in regulating the stress response induced by drug therapy by binding to a variety of signaling proteins. ANXA6 can promote network formation and drug resistance of gastric cancer cells by activating FAK-YAP signaling in the extracellular matrix. The peritoneal metastasis mouse model shows that CAF-EV induces peritoneal tumor resistance, while inhibition of FAK-YAP effectively attenuates gastric cancer drug resistance in vitro and in vivo [96]. This evidence suggests that FAK-YAP signaling pathway is also an important pathway associated with drug resistance through ANXA6.

## ANXA6 and metabolic reprogramming

The ability of cancer cells to alter their metabolism is one of the main mechanisms involved in the rapid progression of solid tumors and one of the hallmarks of malignancy. The metabolic phenotype dependence evolves during different stages of cancer development: Early tumor growth requires nutrient uptake and biosynthesis; Other subtypes of particular metabolic requirements emerge during local infiltration; Due to treatment resistance, new metabolic phenotype dependence arises during late progression, especially during metastasis [97]. The metabolic changes caused by ANXA6 are mainly abnormal cholesterol metabolism [11, 98] and glycolysis flux [99, 100].

In cancer cells, cholesterol metabolism is frequently reprogrammed. Targeting cholesterol metabolism as a new therapeutic method has attracted increasing attention [87]. Exogenous cholesterol directly activates the tumorigenic Hedgehog pathway, and endogenous cholesterol induces the mammalian target of rapamycin C1 (mTORC1) signaling pathway. Lipid rafts composed of cholesterol are the primary platform for cancer signaling regulation, and chelating membrane cholesterol is also an effective anti-cancer strategy [11]. ANXA6 depletion in mesenchymal-like TNBC cells is associated with reduced mitochondrial fatty acid oxidation and lipid droplet accumulation. The absence of ANXA6 transforms the lipogenic phenotype of these cells to a lysogenic phenotype [100]. On the other hand, morphological analysis of liver sections from ANXA6-KO mice showed that ANXA6 deficiency significantly decreased the number of hepatic fat droplets [101]. ANXA6 has been linked to the control of adipose composition and adiponectin release from adipocytes, which may contribute to obesity [102, 103]. Moreover, some adipokines (such as leptin, resistin, and visfatin) are overproduced in obesity and extensively involved in different stages of cancer by promoting cellular glucose and lipid metabolism [98]. In addition, Cairns et al. [104] demonstrated that ANXA6-KO mice on a high-fat diet (HFD) gained less weight than controls, displayed reduced adiposity and failed to downregulate hepatic gluconeogenesis, despite similar insulin levels and insulin signaling activity as controls. Above all, ANXA6 silencing may enhance fatty acid oxidation metabolism and reduce lipid accumulation.

In terms of glucose metabolism, Cairns et al. [104] observed increased glycogen storage in the liver of HFD- and chow-fed ANXA6-KO animals, together with an inability to reduce glucose production after insulin exposure in ANXA6-depleted HuH7 hepatocytes, this implicates ANXA6 contributing to the regulation of hepatic glucose metabolism. Coincidentally, Alvarez et al. [105] found that ANXA6-KO mice induce acute proliferation and metabolic stress after partial hepatectomy (PHx) and exhibit low survival rates. This is associated with an irreversible and progressive decline in blood glucose levels. Although exogenous glucose administration or restoration of hepatic ANXA6 expression rescued the survival of ANXA6 knockout mice after PHx, persistent hypoglycemia resulted from impaired alanine-dependent gluconeogenesis in ANXA6-deficient hepatocytes. Cytoplasmic solute carrier family 38 member 4 (SLC38A4) could not recycle to sinusoidal plasma membranes of ANXA6-deficient hepatocytes 48 h after PHx, impairing alanine uptake and thus glucose production as a possible mechanism [105]. In addition, downregulation of ANXA6 in TNBC cells typically attenuates mitochondrial respiration, glycolysis flux, and cellular ATP production, further leading to a quiescent metabolic phenotype [100]. In cells lacking ANXA6, mitochondria are fragmented, and the mitochondrial membrane potential is reduced [106]. Therefore, the ability to regulate glucose metabolism may be reduced or even lost with the silencing of ANXA6, and ANXA6 is an essential protein involved in glucose metabolism.

Carbohydrate and lipid metabolism ensuring a constant energy supply is a prominent feature of highly proliferating cancer cells. A continuous energy supply supports highly proliferating cancer cells to adapt to hypoxic environments and protects them from oxidative stress. This rewired metabolic property is usually the result of epigenetic alterations in cancer cells. In contrast, the epigenetic landscape of cancer cells is also determined by their different metabolic settings. Such metabolic and epigenetic interactions have great potential for the development of effective anti-cancer therapeutic strategies.

## Other tumor-associated phenotypes

ANXA6 and aging: Differential expression of ANXA6 is associated with vascular disease. ANXA6 was found to be highly expressed, and enhancer of zeste homolog 2 (EZH2) was lowly expressed in Ang II-induced aging models of blood smooth muscle cells. Knockdown of ANXA6 or overexpression of EZH2 to inhibit Ang II-induced ROS can inhibit cellular senescence. In addition, reducing Ang II induces G1 arrest and increases G2 phase cells. ANXA6 overexpression has the opposite effect. EZH2 regulates ANXA6 promoter H3K27me3 modification, inhibits ANXA6 expression, attenuates Ang II-induced senescence in blood smooth muscle cells, and inhibits the progression of an abdominal aortic aneurysm (AMA) [107].

ANXA6 and membrane repair: Cancer cells can reach distant tissues through migration and invasive processes. Cell membrane damage may inhibit cancer metastasis. Therefore, inhibition of membrane repair signaling may inhibit cancer metastasis [63]. ANXA6 is a protein involved in plasma membrane repair. MCF7 cells counteract lipopeptide-induced membrane permeabilization by activating the plasma membrane repair system (extracellular Ca^2+^-triggered ANXA6), and the cytotoxicity of lipopeptides can be increased by knocking down ANXA6 [108]. After skeletal muscle injury, ANXA6 treatment can prevent acute muscle injury in vitro and in vivo and reduce serum creatinine kinase levels [109]. During membrane repair, translocation of ANXA6 from the cytoplasm to the damaged cell membrane prevents excessive Ca^2+^ from entering cells and causing damage [60–62]. These evidences suggest that ANXA6 may influence cancer therapy through membrane repair mechanisms.

## Summary and prospects

ANXA6 is differentially expressed in various tumors and is widely involved in multiple phenotypes, including tumor formation, progression, drug resistance, metabolic reprogramming, and other related phenotypes. However, there are few studies on ANXA6 in tumors, and its mechanism of action in many cancers still remains unclear. In the future, the continuous changes of ANXA6 in the different stages of tumorigenesis and the specific mechanism of chemotherapy resistance are worthy direction to explore. The information reviewed in this article may expand researchers’ understanding of ANXA6 and contribute to the future development of ANXA6-based treatment strategies for cancer patients.
